# Cerebrovascular complications in pediatric intensive care unit

**DOI:** 10.4103/0972-5229.74171

**Published:** 2010

**Authors:** Anil Sachdev, Rachna Sharma, Dhiren Gupta

**Affiliations:** **From:** Department of Pediatrics, Sir Ganga Ram Hospital, New Rajinder Nagar, New Delhi, India; 1 Department of Pediatrics, Dr. B. L. Kapoor Memorial Hospital, Pusa Road, New Delhi, India

**Keywords:** Anticoagulant therapy, cerebrovascular accident, childhood stroke, pediatric intensive care unit, stroke, thrombolytic therapy

## Abstract

Cerebrovascular complications are being frequently recognized in the pediatric intensive care unit in the recent few years. The epidemiology and risk factors for pediatric stroke are different from that of the adults. The incidence of ischemic stroke is almost slightly more than that of hemorrhagic stroke. The list of diagnostic causes is increasing with the availability of newer imaging modalities and laboratory tests. The diagnostic work up depends on the age of the child and the rapidity of presentation. Magnetic resonance imaging, computerized tomography and arteriography and venography are the mainstay of diagnosis and to differentiate between ischemic and hemorrhagic events. Very sophisticated molecular diagnostic tests are required in a very few patients. There are very few pediatric studies on the management of stroke. General supportive management is as important as the specific treatment. Most of the treatment guidelines and suggestions are extrapolated from the adult studies. Few guidelines are available for the use of anticoagulants and thrombolytic agents in pediatric patients. So, our objective was to review the available literature on the childhood stroke and to provide an insight into the subject for the pediatricians and critical care providers.

## Introduction

Cerebrovascular accident in children is more common than was once recognized. It is typically associated with an underlying anatomic anomaly or a systemic disease. The cerebrovascular complications are due to any abnormality of the brain resulting from a pathologic process of the blood vessels, e.g., occlusion of the lumen by a thrombus or embolus, rupture of the vessel, any lesion or altered permeability of the vessel wall and increased viscosity or other change in quality of blood.

This review article is presented since there are few pediatric studies on childhood stroke and clear guidelines are not available to deal with such patients in the pediatric intensive care. A search of publications listed in the electronic databases Pubmed and OVID was conducted using the keywords like stroke, childhood stroke, cerebrovascular accident, thrombolytic therapy and anticoagulant therapy. We preferred to select studies on pediatric stroke particularly and also studies on adults with reference to children. The relevant cross-references from the eligible articles were also searched manually. Some articles were also obtained manually from National Medical Library, New Delhi. The selection of studies was based on study title initially, followed by the abstract and full body text. Two hundred studies were selected on the basis of study title. This was reduced to 130 after abstract search and 65 full text articles were selected and read.

## Epidemiology

It is a general impression that childhood cerebrovascular diseases are more often underdiagnosed as compared to that of the adult population. The overall average annual incidence rate for children through 14 years of age was 2.52/100,000/year.[1] Broderick *et al*,[2] found an incidence of 2.7 cases/100,000/year, similar to the figure reported previously by Schoenberg and colleagues.[1] In the Canadian Pediatric Ischemic Stroke Registry,[3] incidence of arterial and venous occlusion is estimated to be 1.2/100,000 children/year.

## Risk Factors

Strokes in children occur in conjunction with intracranial infection, arteriovenous malformations (AVMs) or with occlusive vascular diseases secondary to cardiac disease, hematological and metabolic disorders. Central nervous system infections and trauma remain the major causes of stroke in children. Despite extensive evaluation, an etiologic factor or associated conditions remain undetermined in 20–50% of all stroke patients [Table 1].[4–6] The incidence of stroke in pediatric cardiac patients is 4% with 75% occurring within the first two years of life.[7]

**Table 1:** Risk factors for pediatric cerebrovascular disease

Cardiac causes	Hematologic disorders and coagulopathies
Congenital heart disease	Hemoglobinopathies (sickle cell anemia)
Ventricular septal defect	Immune thrombocytopenic purpura
Atrial septal defect	Thrombotic thrombocytopenic purpura
Patent ductus arteriosus	Thrombocytosis
Aortic stenosis	Polycythemia
Mitral stenosis	Disseminated intravascular coagulation
Coarctation	Leukemia or other neoplasm
Cardiac rhabdomyoma	Congenital coagulation defects
Complex congenital heart defects	Oral contraceptive use
Acquired heart disease	Pregnancy and the postpartum period
Rheumatic heart disease	Antithrombin III deficiency
Prosthetic heart valve	Protein S deficiency
Libman–Sacks endocarditis	Protein C deficiency
Bacterial endocarditis	Congenital serum C2 deficiency
Cardiomyopathy	Liver dysfunction with coagulation defect
Myocarditis	Vitamin K deficiency
Atrial myxoma	Lupus anticoagulant
Arrhythmia	Anticardiolipin antibodies
Systemic vascular diseases	Structural anomalies
Systemic hypertension	Arterial fibromuscular dysplasia
Volume depletion or systemic hypotension	Agenesis or hypoplasia of the internal carotid or vertebral arteries
Hypernatremia	Arteriovenous malformation
Superior vena cava syndrome	Hereditary hemorrhagic telangiectasia
Diabetes	Sturge–Weber syndrome
Vasculitis	Intracranial aneurysm
Meningitis (bacteria, tuberculosis, fungi)	Trauma
Systemic infection	Fat or air embolism
Systemic lupus erythematosus	Foreign body embolism
Polyarteritis nodosa	Carotid ligation (e.g., ECMO)
Granulomatous angiitis	Vertebral occlusion following abrupt cervical rotation
Takayasu’s arteritis	Child abuse
Rheumatoid arthritis	Post-traumatic arterial dissection
Dermatomyositis	Blunt cervical arterial trauma
Inflammatory bowel disease	Arteriography
Drug abuse (cocaine, amphetamines)	Post-traumatic carotid cavernous fistula
Hemolytic-uremic syndrome	Coagulation defect with minor trauma
Vasculopathies	Amniotic fluid/placental embolism
Ehlers–Danlos syndrome	Penetrating intracranial trauma
Homocystinuria	
Moyamoya syndrome	
Fabry’s disease	
Malignant atrophic papulosis	
Pseudoxanthoma elasticurn	
NADH-CoQ reductase deficiency	
Vasospastic disorders	
Migraine, ergot poisoning, vasospasm with subarachnoid hemorrhage	

**Figure 1:**
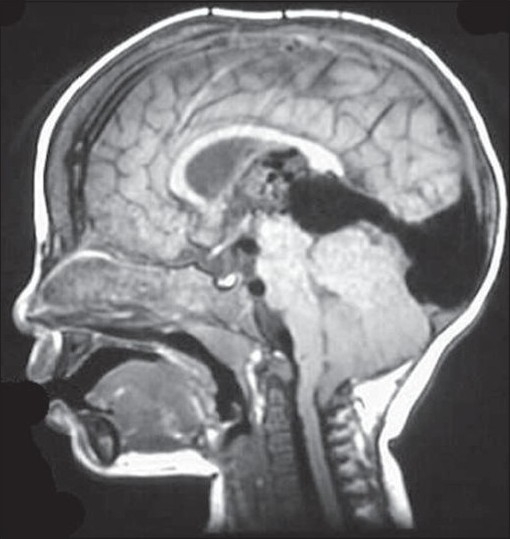
A vein of Galen malformation

Vascular malformations, including AVMs, aneurysms, and cavernous malformations are the most common surgically treatable risk factors for children with hemorrhagic stroke [Figure 1].[8,9] Hematological disorders leading to hyperviscosity syndromes (polycythemia, hyperleukocytosis, and thrombocytosis) can lead to arterial occlusion.[10,11]

There has been a recent recognition of the importance of hypercoagulable states. Antithrombin III, proteins C and S are naturally occurring anticoagulants whose deficiencies are inherited as an autosomal recessive trait.[12,13] Autoimmune disorders may lead to cerebrovascular disease through a vasculitis or by inducing a hypercoagulable state. In systemic lupus erythematosus, neurologic involvement is seen in over 50% of patients.[14]

Several inborn errors of metabolism are associated with cerebral infarction. Homocystinuria, due to a defect in methionine metabolism, may present as a thrombotic syndrome due to endothelial damage and increased platelet aggregation.[15] Stroke-like episodes in a nonvascular distribution are seen in mitochondrial encephalomyopathy with lactic acidemia (MELAS). This recently described disorder is due to a mutation of mitochondrial DNA and a tentative diagnosis can be made by the presence of ragged red fibers in skeletal muscle.[16]

Trauma to the neck may predispose to carotid thrombosis. This can be a blunt injury to the neck, intraoral trauma, falling with a pencil in the mouth, or trauma to the cervical spine (chiropractic manipulation).

Moyamoya disease occurs primarily in the Japanese population, and is characterized by progressive stenosis and occlusion of the cerebral arteries at the Circle of Willis. In response to the stenosis, an abnormal network of small collateral vessels develops, creating the characteristic “puff of smoke” appearance on angiograms. Children with Moyamoya disease present with recurrent transient ischemic attacks as well as acute ischemic stroke. In general, there is a progressive neurologic deterioration that is characterized by significant impairment of motor and cognitive function.[17,18] The majority of cases are idiopathic. Moyamoya syndrome is a disorder with a similar angiographic appearance to Moyamoya disease but is secondary to several slowly progressive occlusive cerebral vasculopathies, such as sickle cell disease, postradiation vasculopathy and neurofibromatosis.

Newborn illnesses that increase the risk of sino-venous thrombosis include asphyxia, dehydration, sepsis, and head and neck disorders, including meningitis. In children, acute illnesses like sepsis or dehydration are present in one-third of the cases of sinus thrombosis.[19] Head trauma or cranial surgery may damage dural sinuses, triggering thrombosis. Chronic systemic inflammatory diseases are the underlying risk factor in 60% of the patients with cerebro-sinovenous thrombosis (CSVT).[20,21]

## Pathophysiology

**Figure 2:**
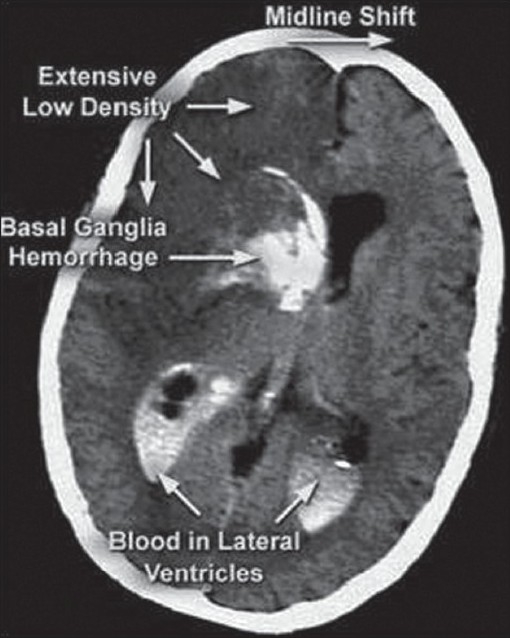
Extensive cerebral haemorrhage

Strokes are broadly classified as either hemorrhagic or ischemic, but may also be caused by decrease in cerebral blood flow.[22] Acute ischemic stroke refers to strokes caused by thrombosis or embolism and accounts for 85% of all strokes. Thrombotic strokes can be divided into large vessel disease, including the carotid artery system, or small vessel disease comprising the intracerebral arteries, including the branches of the Circle of Willis and the posterior circulation. The most common sites of thrombotic occlusion are cerebral artery branch points, especially in the distribution of the internal carotid artery.[23] Cerebral hemorrhage [Figure 2] may occur when an artery in the brain tears or bursts, causing blood to spill out. A hemorrhage often happens without warning. In children, it may result from blood vessel defects like aneurysm or an AVM present since birth. Very rarely, it may occur as a result of high blood pressure.[24]

Loss of perfusion to a portion of the brain initiates the ischemic cascade within seconds to minutes. Within the ischemic cerebrovascular bed, there are two major zones of injury: the core ischemic zone of necrosis and the potentially reversible “ischemic penumbra”. Due to inadequate supply and rapid depletion of oxygen and glucose in the core zone, the neurons and the supporting tissues (glial cells) undergo necrosis. The cells in the penumbra zone suffer mild to moderate ischemia due to the presence of collateral circulation. Restoration of perfusion to ischemic penumbra is the earliest goal of treatment of ischemic stroke.[25]

**Figure 3:**
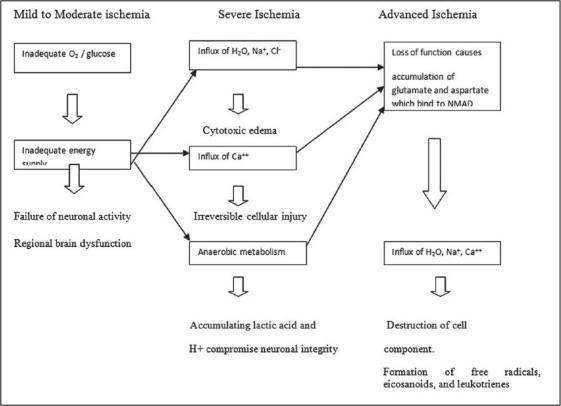
Ischemic cascade of cellular damage (NMAD – N-methyl-d aspartate)

Cellular changes during the ischemic event involve a series of complex metabolic changes. The duration, severity, and the location of focal cerebral ischemia determine the extent of brain function and thus the severity of stroke. Once the blood flow to the neurons diminishes, anaerobic glycolytic pathways are utilized in the affected region and hydrogen ions and lactic acid are produced leading to generation of free radicals, arachidonic acid, nitric oxide and cytokines [Figure 3].[26,27]

## Clinical Manifestation

The signs and symptoms depend on the location and size of the occluded vessel, as well as the patient’s age. Anterior circulation strokes are much more common than posterior, and the left cerebral hemisphere is affected more often than the right.[28]

Most children who have suffered a stroke present with hemiparesis with or without seizures. Seizures at the onset of stroke are relatively frequent in children as compared to adults.[29] A prolonged persistent focal deficit following seizures that is inconsistent with Todd paresis raises the possibility of stroke. Gaze palsy or head turning suggests a large supratentorial infarct. If headache is present, arterial dissection or venous thrombosis is the possible cause. Deterioration in the level of consciousness is common in cerebral hemorrhage, large middle cerebral territory infarcts, and posterior fossa strokes, and is an indication for immediate transfer to an intensive care unit with pediatric neurology and neurosurgery facilities available.[30]

The clinical findings of ischemic and hemorrhagic stroke overlap, so a brain imaging study is mandatory to distinguish ischemic stroke from hemorrhage or other structural brain lesions that may imitate stroke. Hemiplegia, headache, seizure or altered levels of consciousness may all herald a potentially reversible or lethal medical or surgical stroke emergency. The time from onset of symptoms to presentation is very useful diagnostically, for example, arteriopathy is more likely to present with a stuttering onset, and “thunderclap” headaches may be indicative of a subarachnoid hemorrhage.

The chance of hemorrhagic stroke is more if a child presents with headache, nausea, vomiting, coma, hemiparesis, seizures and skin lesions. The clinical presentation of acute ischemic stroke more commonly has associated cranial nerve palsy (VII, IX, X, XII, Horner), visual field defects along with hemiparesis, seizures, headache, ataxia and coma.[24]

Middle cerebral artery occlusions commonly produce contralateral hemiparesis, contralateral hypesthesia, ipsilateral hemianopsia and gaze preference towards the side of the lesion. Agnosia is common, and receptive or expressive aphasia may result if the lesion occurs in the dominant hemisphere. Since the middle cerebral artery supplies the upper extremity motor strip, weakness of the arm and face is usually worse than that of the lower limb.[31]

Anterior cerebral artery occlusions primarily affect frontal lobe function and can result in disinhibition and speech perseveration, producing primitive reflexes (e.g., grasping, sucking reflexes), altered mental status, impaired judgment, contralateral weakness (greater in legs than in arms), contralateral cortical sensory deficits, gait apraxia, and urinary incontinence.[32]

Posterior cerebral artery occlusions affect vision and thought process, producing contralateral homonymous hemianopsia, cortical blindness, visual agnosia, altered mental status, and impaired memory.[33]

Vertebrobasilar artery occlusions are notoriously difficult to detect because they cause a wide variety of cranial nerve, cerebellar, and brainstem deficits.[34] These include vertigo, nystagmus, diplopia, visual field deficits, dysphagia, dysarthria, facial hypesthesia, syncope, and ataxia. A hallmark of posterior circulation strokes is that there are crossed findings: ipsilateral cranial nerve deficits and contralateral motor deficits. This is in contrast to anterior strokes, which produce findings on one side of the body only.

Lastly, lacunar strokes result from occlusion of the small, perforating arteries of the deep subcortical areas of the brain, accounting for 13–20% of all cerebral infarctions. The most common lacunar syndromes include pure motor, pure sensory and ataxic hemiparetic strokes. These infarcts commonly occur in patients with small vessel disease, such as diabetes and hypertension, and do not lead to impairments in cognition, memory, speech, or level of consciousness.[35]

The clinical features of cerebro-sinovenous thrombosis are subtle and present with diffuse neurologic signs and symptoms such as headache, altered sensorium and papilledema in 90% of patients, while seizures are present in 48% and hemiparesis in 17% only.[36,37]

## Laboratory Investigations

The sequence of investigations depends on the clinical presentation and the duration of onset of stroke [Table 2]. Magnetic resonance imaging (MRI) is an ideal method to evaluate neonates, infants, and children with suspected cerebral ischemia. MR angiography can confirm vessel patency and define the vascular anatomy. However, the rapid acquisition time and ease of monitoring make computerized tomography (CT) the ideal imaging technique in an unstable patient or a patient in whom acute intracranial hemorrhage is likely.[38]

**Table 2:** Laboratory investigations in pediatric strok

First line tests (within 24 hours)	Second line tests (within first week if indicated)	Third line tests (Electively)
Magnetic resonance imaging	ECG, ECHO, Carotid Doppler, Holter	HIV, mycoplasm,
Magnetic resonance angiography	Hypercoagulable state	Lyme disease, Cat scratch
Computerized tomography with	Protein C and S (activity, antigen)	Serology
Angiography	Antithrombin III	Cardiac MRI
Conventional angiography	Anticardiolipin antibodies	Transesophageal ECHO
*Other tests*	Antiphospholipid antibodies	Cerebral angiogram
Complete blood counts, ESR	Factor V (leiden) mutation	Muscle biopsy
PT/APTT,	Lupus anticoagulant	Leptomeningeal biopsy
Blood sugar	Rheumatoid factor	DNA study for MELAS
LFT, KFT	Serum aminoacids	Serum homocysteine
Serum electrolytes	Complement profile	
Chest X-ray	Hb electrophoresis	
ANA	Urine for organic acids	
Urinalysis	Serum lactate, pyruvate, ammonia	
Urine drug screen	CSF analysis	
	Lipid profile	

PT prothrombin time, APPT activated partial thromoboplastin time, ANA antinuclear antibody, MELA mitochondrial encephalomyopathy with lactic acidemia

## Management

Acute treatment of cerebral ischemia requires an intensive care unit setting. Treatment should be directed to the underlying cause if it is identifiable. CT or MRI scan of the brain is essential to determine whether stroke is ischemic or hemorrhagic. The management is essentially aggressive supportive care to prevent neurological sequelae.[39] (Table 3)

**Table 3:** Acute medical management of adult and childhood arterial ischemic stroke

Acute therapy	RCP pediatric guidelines[62]	ACCP pediatric guidelines[63]	AHA adult guidelines[40]
Oxygen	Oxygen saturation should be maintained within normal limits	None	Hypoxic patients with stroke should receive supplemental oxygen (Class I, Level of Evidence C)
Temperature	Temperature should be maintained within normal limits	None	It is agreed that sources, if fever, should be treated and antipyretic medications administered to reduce temperature in patients with stroke (Class I, Level of Evidence C)
Glucose	None	None	It is agreed that hypoglycemia should be treated in patient with acute ischemic stroke (Class I, Level of Evidence C)
Blood pressure	None	None	It is generally agreed that patients with markedly increased blood pressure may have their blood pressure lowered. A reasonable goal would be to reduce blood pressure by 15% during the first 24 hours after the onset of stroke. The level of blood pressure that would mandate such a treatment is unknown, but consensus exists that medications should be withheld unless the systolic blood pressure is >220 mmHg or the diastolic blood pressure is >I20 mmHg (Class I, Level of Evidence C)
Decompressive surgery	Early neurosurgery of referral should be considered in children with stroke who have depressed of deteriorating conscious level or other signs of increased intracranial pressure (strong consensus)	None	Decompressive surgery for malignant edema of the cerebral hemisphere may be life saving, but the impact of morbidity is unknown. Both the age of the patient and the side of the infarction (dominant vs. no dominant hemisphere) may affect decisions about surgery. Although the surgery may be recommended for treatment of seriously affected patients, the physician should advise the patient’s family about the potential outcomes, including survival with sever disability. (Class I Ia, Level of Evidence B)
			Decompressive surgical evacuation of a space occupying cerebellar infarction is a potentially life-saving measure, and clinical recovery may be good (Class I, Level of Evidence B)

RCP = Royal College of Physicians; ACCP = American College of Chest Physicians; AHA = American Heart Association

### 

#### Oxygenation

Maintenance of airway, breathing, and circulation are the first priority in any acutely ill child. Studies on oxygenation in childhood arterial ischemic stroke are lacking, but a single consensus guideline from the Royal College of Physicians (RCP) advises that oxygen saturation should be maintained within normal limits. Supplement oxygen by mask or hood should be provided when oxygen saturation is <92%, and endotracheal intubation and mechanical ventilation should be instituted in patients with severe hypoxia and hemodynamic instability.[40,41]

#### Blood pressure

Elevated blood pressure should not be treated very promptly within the first days after ischemic stroke. The ischemic penumbra loses autoregulation, and perfusion is directly linked to the mean arterial pressure. Acute elevations in blood pressure are often transient, and spontaneous declines are common. Overzealous treatment of hypertension following arterial ischemic stroke can convert the ischemic penumbra into an infarct. Reduction of blood pressure greater than 20 mmHg has been associated with worse neurological outcomes and larger infarcts, leading to the suggestion that even extreme cases of hypertension should be reduced by only 15% in the first 24 hours.[42]

If antihypertensive therapy is necessary, agents that have a rapid onset and predictable response should be used. If a child presents with hypotension, prompt treatment with fluid boluses and ionotropic vasopressors should be instituted to maintain mean blood pressure.[43]

#### Temperature

In patients with acute stroke, fever is not uncommon. Fever should be taken care of with prompt use of antipyretics. Even mild elevations in body temperature consistently worsen the neurologic outcome from ischemic insults.[44,45] Induced hypothermia is a potential treatment strategy for adult arterial ischemic stroke and it improves outcomes of neonates with hypoxic ischemic encephalopathy.[46]

#### Euglycemia

There is strong evidence that hyperglycemia and hypoglycemia are associated with poor outcomes in adult arterial ischemic stroke studies.[47] A similar recommendation has been made for pediatric stroke.[48]

Hyperglycemia induces a pro-oxidative and proinflammatory state that can cause direct neuronal toxicity and cerebral edema. It is also responsible for procoagulant state and may further affect penumbral area blood supply. Besides lowering blood sugar, insulin has antioxidant and anti-inflammatory effects. Insulin also improves nitric oxide production and results in improved blood circulation to the ischemic areas.

#### Increased intracranial pressure

Acute management of large-distribution arterial ischemic stroke in children and adults often involves management of increased intracranial pressure secondary to edema and/or mass effect on the ventricles during the first 72–96 hours after a large infarct. The American Heart Association (AHA) suggests decompressive surgery for the seriously affected patients after adequate counseling of the family,[40] while the RCP recommends early neurosurgical referral in children with deteriorating sensorium. Unfortunately, evidence and guidelines in childhood stroke are lacking.[49]

#### Blood transfusion

For children with sickle cell disease, who have ischemic stroke, exchange transfusion is required to reduce hemoglobin S levels to <30% total hemoglobin, and thereafter, regular transfusion (4–6 weekly) to keep hemoglobin S to <20% is required.[50]

## Recommendations for Supportive Therapy After Stroke in Children

The recommendations are discussed below.[38]

### 

#### Class I recommendation

The supportive measures include maintenance of normothermia, normoxemia, normoglycemia and control of systemic hypertension (Class I, Level of Evidence C).

#### Class II recommendation

In children with stroke, it is reasonable to maintain euhydration and treat anemia (Class IIa, Level of Evidence C).

#### Class III recommendations

1. There is no role of supplemental oxygen in the absence of hypoxemia (Class III, Level of Evidence C).
2. There is no evidence suggesting the benefit of prophylactic antiepileptics in the absence of clinical or electrographic seizures in children with ischemic stroke (Class III, Level of Evidence C).
3. Hypothermia should not be used in children with stroke except in the context of a clinical trial as there are no additional data confirming its safety and efficacy (Class III, Level of Evidence C).

#### Thrombolytic therapy

Tissue plasminogen activator (tPA) is used acutely in adult arterial ischemic stroke as a profibrinolytic agent, for lysing intracerebral clots and thereby restoring blood flow to compromised regions of the brain (Table 4). These patients are now routinely given systemic tPA when presenting within 3 hours of stroke onset.[51] A dose of 0.9 mg/kg (maximum 90 mg) is used, with 10% of the dose given as a bolus over the first minute, and the remaining dose given as a 1-hour infusion.

**Table 4:** Antithrombotic management of adult and childhood arterial ischemic stroke

	RCP pediatric guidelines[62]	ACCP pediatric guidelines[63]	AHA adult guidelines[40]
Acute systemic thrombolysis	No specific guideline, but the following comment: “There is currently no evidence to support use of thrombolytic agents such as tissue plasminogen activator (tPA) in the acute treatment of arterial ischemic stroke in children.”	No specific guideline, but the following comment: “The use of thrombolytic agents in children with arterial ischemic stroke, however, has been rare, and the risk/benefit ratio is unknown at this time.”	Intravenous recombinant tPA (0.9 mg/kg: maximum dose 90 mg) is recommended for selected patients who may be treated within 3 hours of onset of ischemic stroke (Class I, Level of Evidence A)
Acute intra-arterial thrombolysis	None	None	Intra-arterial thrombolysis is an option for treatment of selected patients who have major stroke of <6 hours duration because of occlusions of the MCA and who are not otherwise candidates for intravenous recombinant tPA (Class I, Level of Evidence B)
Acute, nonthrombolytic management of idiopathic arterial ischemic stroke	Aspirin (5 mg/kg/day) should be given once there is radiological confirmation of arterial ischemic stroke, except in patients with evidence of intracranial hemorrhage on imaging and those with sickle cell disease (strong consensus)	For children with arterial ischemic stroke, we suggest treatment with UFH or LMWH for 5–7 days and until cardioembolic stroke or vascular dissection has been excluded (grade 2C)	The oral administration of aspirin (initial dose 325 mg) within 24–48 hours after stroke onset is recommended for treatment of most patients (Class I, Level of Evidence A)

RCP = Royal College of Physicians; ACCP = American College of Chest Physicians; AHA = American Heart Association; MCA = Middle cerebral artery; UFH = Unfractionated heparin; LMWH = Low–molecular-weight hepain

There is currently no evidence to support the use of thrombolytic agents such as tPA in the acute treatment of pediatric arterial ischemic stroke. A prospective study by Browne *et al*,[52] using 0.5 mg/kg/hour systemic recombinant tPA for 6 hours concurrently with heparin (10 U/kg/hour) and fresh frozen plasma supplementation prior to recombinant tPA infusion, reported complete resolution of thrombosis in 13 of 20 children (65%), partial resolution in 4 patients (20%), and no response in 3 patients (15%). Zenz *et al*,[53] reported a dose of 0.5 mg/kg/hour for the first hour followed by 0.25 mg/kg/hour until clot lysis occurred or treatment had to be stopped because of bleeding complications. Complete clot lysis was achieved in 16 of 17 pediatric patients within 4–11 hours after the start of treatment. At this time, there is no evidence to suggest that there is an advantage of local over systemic thrombolytic therapy in children with thrombotic complications.[54] Thrombolytic therapy has been reported to have significant bleeding complications in children (68%) and blood transfusion is required in 39%.[55] Before thrombolytic therapy is used, the correction of other concurrent haemostatic problems such as associated thrombocytopenia or vitamin K deficiency is advisable. In case of major bleeding, discontinuation of thrombolytic agent and administration of cryoprecipitate and other blood components are needed.[38]

## Recommendations for Thrombolytic Therapy for Childhood Stroke

The recommendations are discussed below.[38]

### 

#### Class II recommendation

In children with CVST, thrombolytic therapy with tPA can be considered (Class IIb, Level of Evidence C).

#### Class III recommendation

Thrombolytic therapy is not recommended for children with acute ischemic stroke outside a clinical trial (Class III, Level of Evidence C). However, there was no consensus about the use of tPA in older adolescents who otherwise meet standard adult tPA eligibility criteria.

#### Antiplatelet aggregation

The Joint Guideline Statement from the AHA and the American Academy of Neurology (AAN) recommends that aspirin should be given within 24–48 hours of stroke onset in most patients (Level of Evidence grade A).[40] The administration of aspirin as an adjunctive therapy, within 24 hours of the use of thrombolytic agents, is not recommended (grade A). Aspirin should not be used as a substitute for other acute interventions, especially t-PA (grade A). A low dose of 1–5 mg/kg/day for platelet aggregation inhibition has been proposed.[56] Pediatric doses of aspirin are not based on studies of the effect on platelet function in pediatric patients.

Ticlopidine and clopidogrel selectively inhibit ADP-induced platelet aggregation, via the inhibition of the P2Y _12_receptor. The antiplatelet effect of ticlopidine and clopidogrel is additive to that of aspirin.[57] There has been no reported use in children, and dosage recommendations are unknown.

## Recommendations for Aspirin Use in Children with Stroke

The recommendations are discussed below.[38]

### 

#### Class II recommendations

1. To prevent recurrence of acute ischemic stroke in children, aspirin can be used, except in cases where the underlying cause of stroke is sickle cell disease, severe hypercoagulable disorder and all those children who are at increased risk for embolization (Class IIa, Level of Evidence C).
2. A dose of 3–5 mg/kg/day is adequate for stroke prevention in children (Class IIa, Level of Evidence C). If dose-related side effects occur, then, a dose reduction to 1–3 mg/kg may be considered (Class IIb, Level of Evidence C).
3. In children taking aspirin for stroke prevention, it is reasonable to vaccinate for varicella and annually with influenza vaccine to reduce the risk of Reye’s syndrome (Class IIa, Level of Evidence C). During the period of varicella and influenza infection, it is reasonable to withhold aspirin (Class IIa, Level of Evidence C).

#### Anticoagulants

Heparins, including unfractionated heparin (UFH) and low–molecular-weight heparin (LMWH), are the principal agents used in the acute phase of anticoagulant therapy for arterial ischemic stroke. Based on the high rates of prothrombotic abnormalities, cardioembolic stroke and vascular dissection in childhood arterial ischemic stroke, American College of Chest Physicians’ (ACCP) guidelines[58] recommend treatment with UFH or LMWH for 5–7 days. However, this recommendation remains controversial, and the RCP recommends initial treatment of childhood arterial ischemic stroke with aspirin 5 mg/kg until an indication for anticoagulation is found.[62] Parenteral anticoagulants should not be prescribed until a brain imaging study has excluded the possibility of a primary intracranial hemorrhage.[59] UFH doses are age dependent, with younger children requiring greater average doses (20 U/kg/hour) than older children and adults; similar observations have been made for LMWHs. Bolus dosing of UFH is typically avoided in arterial ischemic stroke. In children, heparin therapy is monitored most accurately by antifactor Xa activity. For UFH, the therapeutic range is 0.35–0.7 antifactor Xa activity U/ml, whereas for LMWH, the therapeutic range is 0.5–1.0 U/ml. Side effects of UFH include bleeding, osteoporosis, and heparin induced thrombocytopenia.[60] Burak *et al*,[61] administered enoxaparin to eight children with stroke and concluded that the LMWH was a safe and effective alternative to heparin for children. Anticoagulants also are being explored as an adjunct to thrombolytic therapy.

## Recommendations for LMWH in Children with Stroke

The recommendations are discussed below.[38]

### 

#### Class I recommendations

For long-term anticoagulation, LMWH can be used in all children with substantial risk of recurrent cardiac embolism, CVST, and selected hypercoagulable states (Class I, Level of Evidence C).

#### Class II recommendations

The administration of LMWH or UFH may be considered in children for up to 1 week after an ischemic stroke, pending further evaluation to determine the cause of the stroke (Class IIb, Level of Evidence C).

## Recommendations for the Use of Warfarin in Children with Stroke

The recommendations are discussed below.[38]

### 

#### Class II recommendations

For long-term anticoagulation, warfarin can be used in all children with substantial risk of recurrent cardiac embolism, cervico-cephalic arterial dissection, CVST, and selected hypercoagulable states (Class IIa, Level of Evidence C).

#### Surgical intervention

Surgical decompression has been reported in children presenting in coma with large ischemic middle cerebral infarcts, which are almost always fatal if managed conservatively. Similar intervention is required for a large cerebellar infarction leading to brain stem compression and hydrocephalus.[38]

## Prevention of Stroke

The mechanisms and risk factors for arterial ischemic stroke in children are not well understood at present. The primary prevention is well established in few well-defined causes of arterial ischemic stroke, such as sickle cell disease, congenital cardiac lesions and hypercoagulable disorders.

1. In sickle cell disease, all the children between 2 and 16 years should have Transcranial Doppler study once in a year (Class IIa, Level of Evidence B). Borderline and mildly abnormal studies may be repeated in 3–6 months. Children with abnormal results should receive periodic transfusions to reduce the percentage of sickle hemoglobin (Class I, Level of Evidence A).[38]
2. All children with congenital heart lesions, especially complex heart lesions, should be repaired both to improve cardiac function and to reduce the subsequent risk of stroke (Class I, Level of Evidence C).[38]
3. Children with hypercoagulable disorders are at a risk of stroke only in the setting of an additional risk factor. However, it is reasonable to evaluate for elevated serum homocysteine levels and the more common prothrombotic states, and to start folate, vitamin B6, or vitamin B12 if homocysteine is found to be elevated.[38]

## Rehabilitation Techniques

Early evaluation of physical and cognitive disability is the key to prevent avoidable complications and to plan rehabilitation, which should involve a multidisciplinary team.[62–64] Constraint therapy may be adapted for children and appears to be associated with improved function of a hemiparetic hand. Improvement occurs over a prolonged period of time, and late deterioration is rare. The child and family should be encouraged to express their main concerns about re-integration to the home, community and school environments and to have these concerns addressed.[65,66]

## Outcome, Morbidity and Mortality

Between 20 and 40% of children die after a stroke.[67–72] The mortality is higher for the hemorrhagic (about a third) than for ischemic stroke (up to 20%, with about half related to the underlying systemic illness rather than the stroke itself). Death during the acute phase is predicted by the level of consciousness on admission. Recurrent stroke occurs in 6–15% of children and mortality is higher in this group.[71,73,74] Intractable intracranial hypertension is a major predictor of poor outcome in patients with large intracerebral or intracerebellar hemorrhage and massive hemispheric or cerebellar infarction. Between 50 and 80% of surviving children have neurological sequelae, most commonly hemiparesis.[70,71,75–79] Neurological outcome appears to be better for those with hemorrhage, CVST, and posterior circulation stroke.[72,80,81] Other problems include neuropsychological deficits, poor attention, behavioral problems, and poor quality of life.[82–86] Predictors of poor neurological, cognitive, and behavioral outcome include systemic disease, multiple risk factors, infarct size, cortical involvement, thromboembolism, and Moyamoya.[87–89]

## Our Experience at Sir Ganga Ram Hospital

In the year 2009, from January to December, there were 794 admissions in the 12-bedded PICU. Of these, 135 cases had primary non-traumatic central nervous system ailment and only 15 cases presented with stroke. There were nine patients with arterial ischemic stroke and six had hemorrhagic stroke. Central nervous system infection was the predominant risk factor for ischemic stroke in six patients, two had severe dehydration and electrolyte imbalance, and one each with cyanotic heart disease and L-asparaginase induced stroke. There were three cases of hemorrhagic stroke due to malignancy and one each of AVM, middle cerebral artery aneurysm and late hemorrhagic disease of the newborn. Neuroimaging was done in all cases for the diagnosis and to differentiate ischemic from hemorrhagic stroke. All patients received supportive therapy, LMWH and aspirin for ischemic stroke while interventional neurosurgical procedures were done in three cases of hemorrhagic stroke. Thrombolytic therapy was not used in any case.

## Conclusions

Intensive supportive management and rehabilitation techniques are critical components in the management of children with stroke to optimize outcomes. The pathophysiology and outcomes of adult stroke differ significantly from those in children. The therapeutic management currently remains similar, largely because of the paucity of evidence from devoted pediatric observational studies and clinical trials. The use of anticoagulants and thrombolytic agents has been insufficiently investigated in childhood stroke, resulting in a lack of guidance. Dedicated pediatric studies are critically necessary to establish a key evidence for the multifaceted management in childhood stroke.
